# Unmet oral health needs and barriers to dental services among socially marginalized youth: a scoping review

**DOI:** 10.3389/froh.2025.1521753

**Published:** 2025-03-12

**Authors:** Pranav Vaishampayan, Jaskaran Singh Beniwal, Piotr Wilk, Sarah McLean, Abbas Jessani

**Affiliations:** ^1^ Department of Epidemiology and Biostatistics, Schulich School of Medicine and Dentistry, Western University, London, ON, Canada; ^2^ Department of Dentistry, Schulich School of Medicine and Dentistry, Western University, London, ON, Canada; ^3^ Department of Anatomy and Cell Biology, Schulich School of Medicine and Dentistry, Western University, London, ON, Canada

**Keywords:** dental health services, dental care, marginalized, youth, unmet needs, barriers

## Abstract

**Objectives:**

Barriers limiting access to oral health significantly impact dental service utilization among socially marginalized youth, often resulting in unmet needs and poor oral health outcomes. Identifying and understanding these barriers is critical to inform the development of strategies to enhance oral healthcare access for this vulnerable population. This review examines the barriers restricting access to oral healthcare and unmet dental needs among socially marginalized youth worldwide, offering insight to guide the development of targeted interventions.

**Methods:**

A comprehensive search was performed across electronic databases, including Embase, MEDLINE (Ovid), Scopus, and the Cochrane Library. Two independent reviewers screened all primary studies, irrespective of publication year, to identify relevant research on barriers to care and unmet oral health needs among socially marginalized youth. Primary studies addressing barriers to oral healthcare access and unmet needs were included, with no restrictions on publication date. Studies published in languages other than English were excluded. Any discrepancies identified during the screening process were resolved through consensus. The CLARITY tool was utilized to evaluate the risk of bias in the included studies.

**Results:**

Of the 484 studies identified, six quantitative and one qualitative study met the inclusion criteria. The review identified multiple barriers such as financial constraints, structural impediments, and psychological factors that inhibit access to dental care facilities among socially marginalized youth. Three studies were conducted in the United States, two in Australia, and one each in the United Kingdom and Kenya. Among the identified barriers, four studies reported financial constraints and structural and logistical challenges, respectively while one study reported psychological barriers to dental care. A high prevalence of unmet needs such as dental caries and periodontal diseases, was observed within this demographic. The unmet dental needs identified in the included studies encompassed dental caries (*n* = 3), missing teeth (*n* = 2), periodontal diseases (*n* = 1), tooth pain (*n* = 1), and dental infections (*n* = 1). However, small sample sizes and lacking in robust study design limit the findings' generalizability, emphasizing the need for more diverse studies on oral health outcomes in socially marginalized youth.

**Conclusion:**

This scoping review identified critical research gaps in regards to access to oral health and dental service utilization among socially marginalized youth. Oral health initiatives are warranted to reduce oral health inequalities among socially marginalized youth.

**Systematic Review Registration:**

https://doi.org/10.17605/OSF.IO/T82D3.

## 1 Introduction

The progression from adolescence to adulthood, also known as “youth”, is characterized by numerous changes that may significantly influence individuals' health and overall welfare. These changes encompass personal, psychological, and social development, including academic achievement, employment acquisition, attaining financial independence, and avoiding involvement with the criminal justice system (1).

Youth can be described as the transitional phase between childhood and adulthood, characterized by a continuum of developmental changes rather than rigid age-based boundaries or specific milestones—for example, engagement in employment or sexual activity initiation (2). The United Nations Department of Economic and Social Affairs (UNESDA) defines youth as individuals typically aged between 15 and 24 years, yet acknowledges the variability of this classification across member states (3). Alternative age brackets, such as 18–30, have also been proposed by institutions such as Statistics Canada, highlighting the diverse perspectives on what age group classifies as youth (3).

A distinct subgroup of youth, identified as socially marginalized youth, experiences additional obstacles in their progression to adulthood. These impediments include but are not limited to lower family income, enduring struggles with substance abuse, and the inability to complete their education (1). Additionally, these impediments also cause a significant burden on their health, including oral health. This subgroup could be disproportionally comprised of recent immigrants, Indigenous peoples, individuals experiencing homelessness, people living with HIV, sexual minorities, and those with low socioeconomic status.

Some common oral health conditions reported by socially marginalized youth are tooth pain, gingivitis, dental caries, periodontal diseases, and dental erosion (4, 5). Furthermore, Johansson and Östberg (6) highlighted that poor oral health among socially marginalized youth is often due to negative past experiences, dental anxiety, dental trauma, and pain associated with dental treatments. Consequently, there exists a notable underutilization of dental care services in this vulnerable population, underscoring the importance of addressing the barriers that impede access to oral care services.

Studies indicate that various socio-economic characteristics including financial limitations, lack of insurance, cultural and language differences, geographical constraints, and psychological factors, function as barriers that restrict the accessibility of socially marginalized youth to essential oral healthcare services. For instance, Sharma and Basnet (7) reported that youth with low socioeconomic status exhibited lower utilization of dental care services. Furthermore, cost and geographical proximity are also reported as a significant determinant of dental care utilization. Approximately 25% of individuals aged 18 and above reported not visiting a dentist due to the inability to afford services (8, 9). For example, Wiener (10) highlighted the limited access to dental care services among Indigenous youth due to extended travel times and reliance on external assistance for transportation. Additionally, Hill et al. (11) reported that participants identifying themselves as Alaska Native, American Indian, Native Hawaiian, or other Pacific Islander were 1.6 times less likely to receive preventive services, such as dental cleaning, compared to their Caucasian counterparts despite having dental insurance. This disparity underscores an inadequate awareness regarding oral care practices among socially marginalized youth.

Furthermore, literature highlights that barriers such as dental anxiety and gender-based discrimination, particularly among transgender and gender nonbinary individuals, play a significant role in limiting access to oral healthcare services. These barriers adversely influence their experiences in dental care settings, perceptions of oral health, and likelihood of seeking preventive care, often in contrast to their cisgender counterparts (12–14). For example, Raisin et al. (12) reported that approximately 48% of participants avoided dental visits due to concerns related to their gender identity. The study further highlighted frequent instances of misgendering and the use of incorrect pronouns, which can serve as negative triggers, contributing to a non-inclusive environment. Such experiences exacerbate barriers to dental care for transgender and gender nonbinary individuals, thereby restricting equitable access to oral health services (12).

The presence of such barriers impeding that access contributes to suboptimal utilization of oral care services among socially marginalized youth, resulting in unmet oral health needs and poor oral health status. Finally, unmet needs in this subpopulation may culminate in exacerbated and severe oral health conditions during later life stages, if left untreated. Despite these concerning findings, there is a lack of comprehensive evidence for individual and societal barriers to accessing dental care and oral health service utilization among socially marginalized youth. Therefore, this scoping review aims to analyze the extent of available literature on the unmet oral health needs of socially marginalized youth globally and investigate the breadth of literature available on barriers to accessing oral healthcare among them.

## 2 Methodology

The Joanna Briggs Institute (JBI) Reviewers Manual was utilized to conduct this scoping review (15). This manual offers detailed instructions for authors to adhere to, covering distinct sections dedicated to synthesizing various kinds of evidence pertinent to different types of review inquiries (15). The manual was utilized as a reference resource to address queries concerning the scoping review procedure. Based on the suggestion provided in the JBI Manual, the scoping review protocol was registered with the Open Science Framework, as PROSPERO has specified that scoping reviews do not qualify for registration in their database (15). We adhered to the reporting guidelines outlined in the Preferred Reporting Items for Systematic Reviews and Meta-Analyses extension for Scoping Reviews (PRISMA-ScR) for this review (16, 17). A completed PRISMA-ScR checklist has been provided as Supplementary File 1. Before commencing study screening, a protocol for this scoping review was registered on the Open Science Framework (doi.org/10.17605/OSF.IO/T82D3). The pre-registered protocol contains essential details concerning selection criteria and the extraction of data from the included publications. This step was taken to ensure maximum transparency in the scoping review process and to affirm that our original objectives aligned with our methodology.

### 2.1 Inclusion criteria

This review aimed to identify research articles examining the accessibility of oral health care services among socially marginalized youth and the barriers preventing their utilization of these services. The target population for this review encompassed socially marginalized youth, aged 18–30 irrespective of their oral health status or outcomes related to oral health care. English-language publications from diverse geographic regions were considered, without imposing any limitations based on publication dates. A comprehensive range of methodologies, comprising qualitative, quantitative, and mixed methods approaches, were included in this review.

### 2.2 Exclusion criteria

The following criteria were used to exclude studies while reviewing publications during screening: studies that do not examine the accessibility of oral health services for socially marginalized youth and the factors impeding access to services; studies that document results not related to oral health or oral health care; studies published in a language other than English; and studies for which the full text was unavailable.

### 2.3 Search strategy

P.V. and J.B., in collaboration with a research librarian, formulated the search strategy aimed at identifying relevant literature concerning the accessibility of oral health care services for socially marginalized youth and elucidating the barriers associated with such accessibility. The databases explored were Medline, Embase, Scopus, and Cochrane Library. For an in-depth understanding of our search methodology, refer to Appendix A.

### 2.4 Reference management

All the citations extracted from every database search were transferred to Covidence (2023) for the elimination of duplicate findings While the majority of publications' full texts were accessible online, any unavailable texts were excluded.

### 2.5 Study screening

Two phases of screening were employed to identify pertinent studies. During the initial stage, only the titles and abstracts were assessed, while the subsequent stage involved a thorough review of the full texts. Both screening stages were carried out independently by two reviewers (P.V. and J.B.). Any discrepancies between reviewers were resolved through discussions.

### 2.6 Data extraction

A standardized tool for data extraction (Supplementary File 2) was formulated to facilitate the extraction and comparison of pertinent information across the encompassed studies. Initially, the data extraction tool underwent a pilot phase involving 25% of included studies, following which adjustments were made to ensure comprehensive extraction of all pertinent data. All revisions made have been incorporated into the final version of the data extraction tool (Supplementary File 2). The data extraction process was carried out and validated by both reviewers P.V. and J.B.

### 2.7 Risk of bias assessment of included studies

While scoping reviews typically do not evaluate the risk of bias in the included studies, we considered it essential for our objectives due to the absence of robust study designs. This assessment aimed to ascertain the quality of evidence presented by the included studies. We employed the CLARITY Group's Risk of Bias Instrument for Cross-Sectional Surveys of Attitudes and Practices (CLARITY Group at McMaster University 2021) to evaluate the risk of bias. This instrument was selected for its ease of understanding and ability to provide a comprehensive overview based on five domains (Representativeness of the sample, Adequacy of the response rate, Missing data within completed questionnaires, Conduct of Pilot testing, and established validity of survey instrument). Each criterion is addressed through a question format with four response options: definitely yes (low risk of bias), probably yes (low risk of bias), probably no (high risk of bias), and definitely no (high risk of bias). This instrument was employed because it facilitates the reporting of risk of bias on a domain-specific basis rather than providing an overall single rating.

## 3 Results

### 3.1 Search results

**Figure 1:**
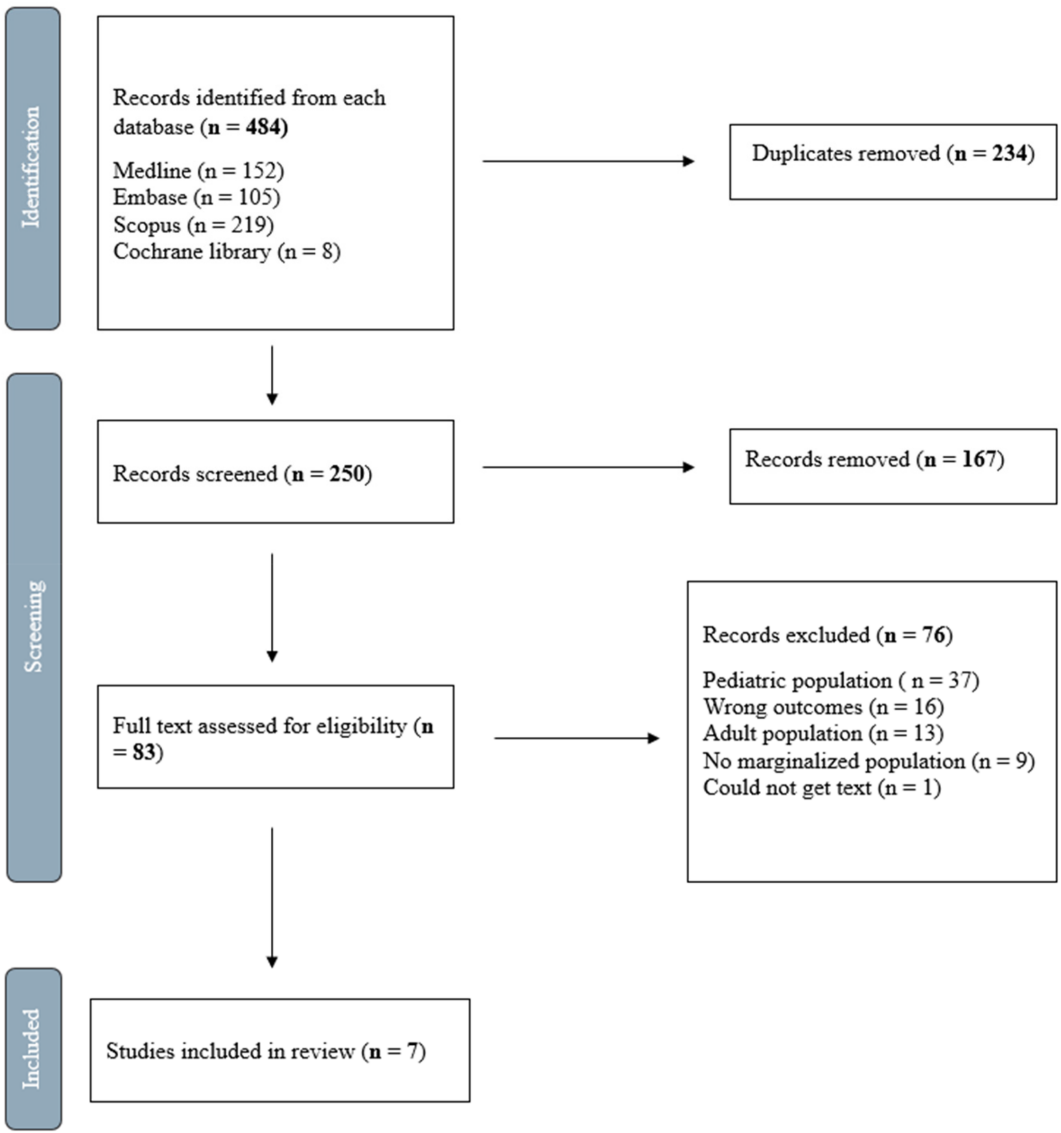
Flow diagram of study selection. Adapted from Tricco et al. (16).

The outcomes of the search and screening process are illustrated in the accompanying figure (Figure 1). It presents the PRISMA flow diagram, outlining the selection of articles included in the review. Following the implementation of the search strategy, a total of 484 studies were identified across various databases: Medline (*n* = 152), Embase (*n* = 105), Scopus (*n* = 219), and Cochrane Library (*n* = 8). Subsequently, 234 duplicate studies were removed, leaving 250 studies eligible for title and abstract screening. From these, 167 studies were excluded, resulting in 83 studies selected for full-text review. The full-text review excluded an additional 76 studies for various reasons, primarily due to the lack of identified youth populations. Finally, seven studies were included in our review that underwent data extraction.

### 3.2 Descriptive characteristics

**Figure 2:**
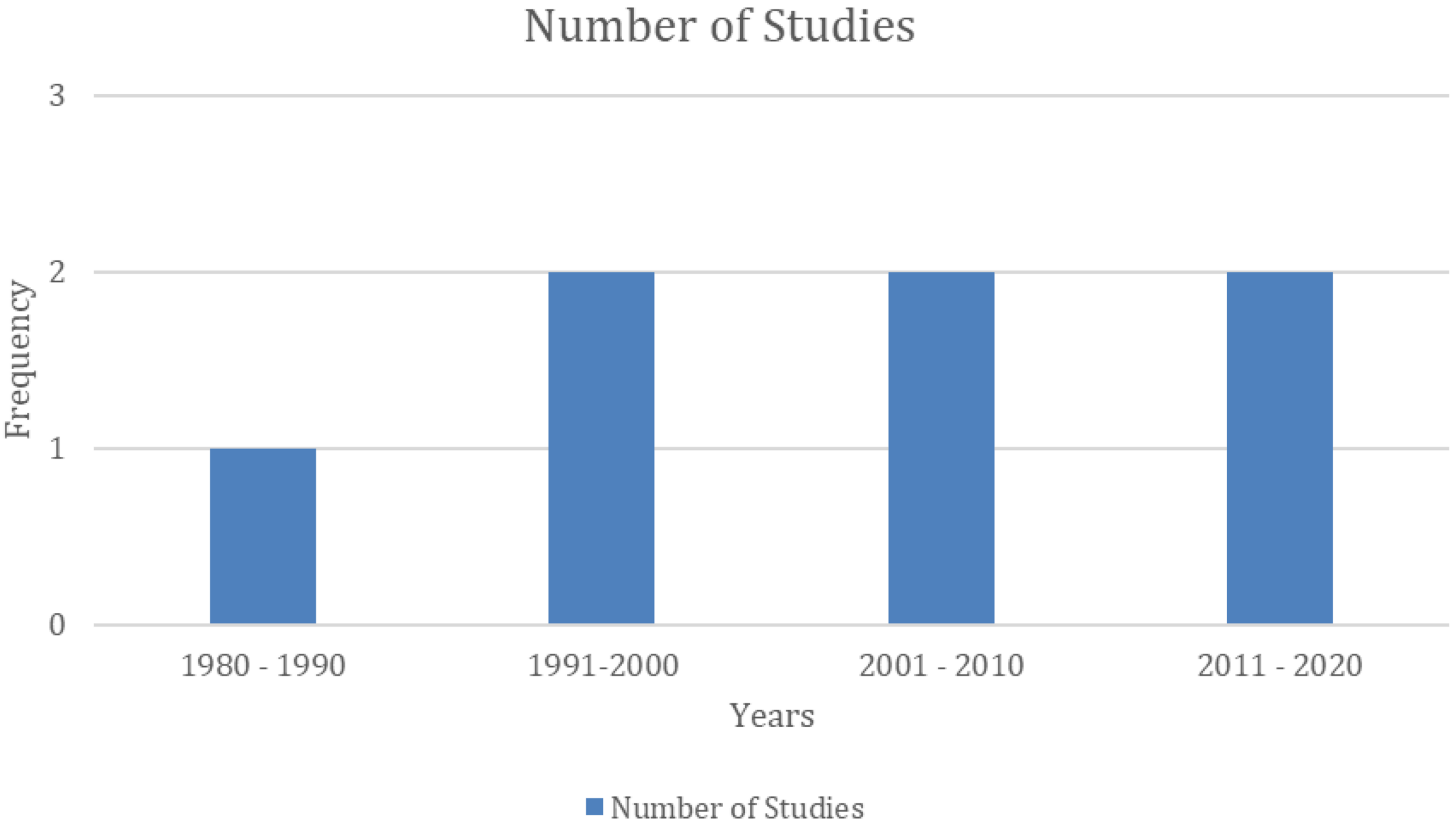
Frequency distribution of studies based on the publication date.

**Figure 3:**
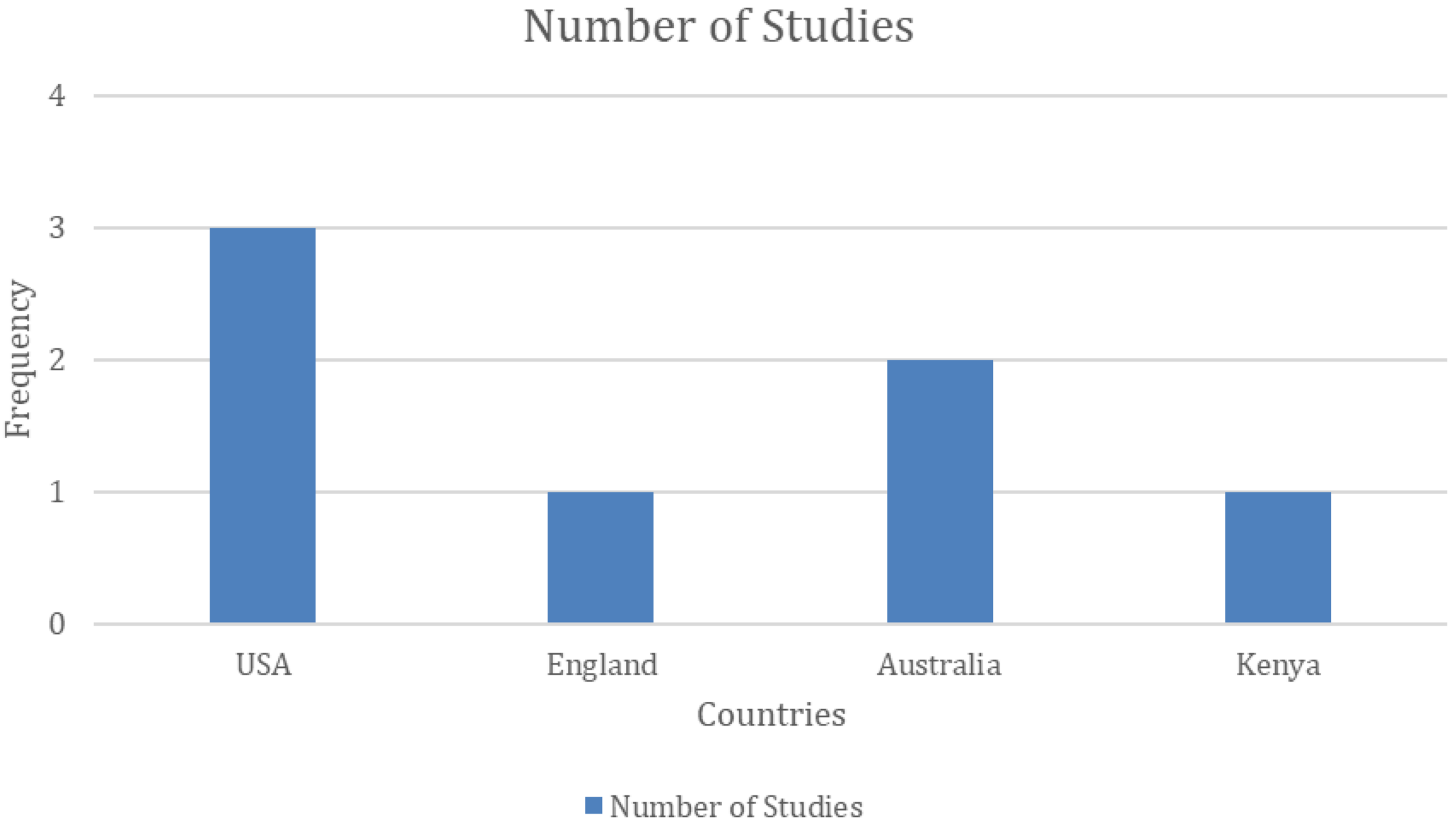
Frequency distribution of studies based on countries of origin.

Figure 2 displays the distribution of all studies included in this review according to their year of publication and Figure 3 illustrates the frequency of countries in which the studies were conducted. The studies were conducted in the United States [*n* = 3; (8, 18, 19)], Australia [*n* = 2; (20, 21)], United Kingdom [*n* = 1; (22)], and Kenya [*n* = 1; (23)]. Notably, all included studies were published in or after 1989 and are observational in design [*n* = 7; (8, 18–23)]. Furthermore, sampling methods included random sampling [*n* = 4; (8, 19, 21, 23)], convenience sampling [*n* = 2; (18, 20)], and snowball sampling [*n* = 1; (22)]. Sample sizes varied, with two studies having less than 100 participants (21, 22), two having between 100 and 500 participants (19, 20), and three studies having more than 500 participants (8, 18, 23). Additionally, marginalization factors reported include racial and ethnic minorities [*n* = 4; (8, 18, 19, 22)], low-income [*n* = 1; (19)], homelessness [*n* = 1; (20)], residence in a rural area [*n* = 1; (23)], and refugee status [*n* = 1; (21)].

### 3.3 Objective 1: unmet oral health needs and patterns of dental service utilization

Table 1 also presents the findings reported by each study regarding unmet needs and oral health services utilization. Six out of seven studies report findings pertaining to this objective (8, 18–21, 23). Out of the six studies, four studies reported on the unmet dental needs of participants (19–21, 23) whereas four reported information regarding patterns of utilization of oral health services (8, 18–20).

**Table 1:** Data extraction table.

Author(s), year	Country	Type of study	Sample size (*n*)	Sampling method	Age range	Marginalization factor	Barriers to care	Patterns of dental visits; unmet needs
Aday and Forthofer, 1992 (18)	USA	Cross-sectional	58,435	Sample of convenience	>18 years vs. 2–17 years	Racial and ethnic minority	No insurance	Individuals residing in metropolitan areas (OR = 1.145), people with private insurance (OR = 1.711), and people who perceived their health as good or very good (OR = 1.260) were more likely to visit a dentist
Chattopadhyay et al., 2003 (8)	USA	Cross-sectional	1,836	Random sampling	18–25 years vs. 25–39 and 40+	Racial and ethnic minority	Cost	Dental visit (%, age): 67% (18–25), 59% (25–39), 37.4% (40+) People with dental insurance (OR = 2.5) and married individuals (OR = 1.7) were more likely to visit a dentist People with low educational backgrounds were less likely to visit a dentist (OR = 0.6)
Cohen et al., 2011 (19)	USA	Cross-sectional	401	Stratified random sample	21–34 years vs. 35–49, 50–64 and 65+ years	Low-income Racial/Ethnic minority	Cost	Dental visits: 87.9% 12% did not visit the dentist within the last two years.
Croucher and Sohanpal, 2006 (22)	UK	Cross-sectional	68	Snowball	18–40 years	Racial/Ethnic minority	High cost Distance Lack of appointments Long waiting time (2 months) (except emergency cases) Dental anxiety: Discomfort in visiting/accepting treatment from a dentist of the opposite gender	Not reported
Manji et al., 1989 (23)	Kenya	Cross-sectional	1,131	Random sampling	15–24- and 25–34-years vs. 35–44, 45–54 and 55–65 years	Rural area	Distance	Lesions involving Enamel: 25–34 years old (9.13 ± 8.97) Dental caries: 15–24 years old (48.8%), 25–34 years old (82.4%)
Smith and Szuster, 2000 (21)	Australia	Cross-sectional	Control = 850 Refugees = 86	Simple random sampling for control	15–24- and 25–34-years vs. 35–44 years	Refugee status	Location	Dental visits No dental visits were observed among 15–24 Iraqi refugees whereas one-third of 25–34 Iraqi refugees visited the dentist. Decayed, missing, filled teeth (DMFT) index (mean ± SD) Decayed: 15–24 years old (4.3 ± 3.9), 25–34 years old (5.0) Missing: 25–34 years old (11 ± 6.2) Filled:15–24 years old (5.8 ± 5.1) DMFT: 15–24 years old (12.6 ± 6.4), 25–34 years old (21.9 ± 7.2)
Stormon et al., 2019 (20)	Australia	Cross-sectional	116	Sample of convenience	16–25 years vs. 23–61 years	Homelessness	Cost Lack of dental clinics Transportation	Self-reported health (%) Excellent/Very good (11%), Good (28%), Fair (32%), Poor (30%). Unmet needs (%) (77%)

Studies reported that unmet needs such as decayed and untreated teeth, periodontal conditions, and xerostomia were observed to be prevalent among the participants (21, 23). Several studies have identified age-related disparities in unmet dental needs. Manji et al. (23) reported an age-associated increase in the prevalence of dental caries, from 48.8% among individuals aged 15–24 to 92.9% in those aged 55–65. Similarly, Stormon et al. (20) observed a lower prevalence of decayed teeth in younger participants (15–25 years) compared to older cohorts (23–61 years). However, Smith and Szuster (21) indicated a higher number of decayed teeth in younger individuals (15–24 and 25–34 years) relative to those aged 35–44. Conversely, Smith and Szuster (21) reported a lower prevalence of missing teeth in younger individuals (25–35 years) compared to older participants (35–44 years), a pattern corroborated by Stormon et al. (20) in participants aged 15–25 compared to older individuals aged 23–61. Additionally, the Decayed, Missing, and Filled Teeth (DMFT) index demonstrated a progressive increase with age. Smith and Szuster (21) reported that participants aged 15–24 had a lower DMFT score (12.6 ± 6.4) than those aged 35–44 (19.9 ± 7.6), although individuals aged 25–34 exhibited a slightly higher DMFT score (21.9 ± 7.2) than those in the 35–44 age group.

Furthermore, Cohen et al. (19) reported that participants aged 21–34 were less likely to report concerns with tooth pain (10.6%), broken teeth or restorations (2.4%), and infections (17%) compared to the 35–49 age group (40.4%, 16.7% and 27%, respectively). This study also reported that participants aged 21–34 were more likely to report periodontal problems (34.6%) and oral conditions such as sores, ulcers, bad taste, and burning sensation (32.3%) when compared to participants aged 35–49 (6.1%, 0.2%).

Regarding patterns of utilization of services, Chattopadhyay et al. (8) reported that dental visits were more frequent in participants aged 18–25 (67%) compared to participants aged 25–39 (59%) and 40+ (37.4%). A similar pattern was observed in the study by Stormon et al. (20) where 25% of participants aged 16–25 visited the dentist in the past 12 months compared to 24% of individuals aged 23–61. Furthermore, certain studies only reported the likelihood of dental visits among participants. For example, Aday and Forthofer (18) reported that males, members of larger families, and individuals without employment were less likely to visit a dentist. Specifically, males older than 18 were less likely to visit a dentist compared to males aged 2–17 years old. Further, unemployed participants and those living in non-metropolitan areas who were 18 years and older were less likely to visit a dentist compared to participants aged 2–17 years who were unemployed and those living in non-metropolitan areas (18). However, participants aged 18 years and older with private insurance and those who perceived their oral health as good exhibited a greater likelihood of a dental visit compared to participants aged 2–17 years with private insurance and those who perceived their oral health as good (18).

### 3.4 Objective 2: barriers restricting access to oral healthcare

Barriers restricting access to oral care services are presented in Table 1. All seven studies (8, 18–23) identified the barriers experienced by participants while accessing oral care. These barriers are classified into three themes: Financial Barriers, Structural and Logistical Barriers, and Psychological Barriers.

#### 3.4.1 Financial barriers

Among reported barriers, cost was determined to be the most commonly reported factor for participants who avoided dental care [*n* = 4; (8, 19, 20, 22)]. Approximately 63% of individuals reported an inability to afford dental care services (20). Moreover, not having any type of insurance also significantly impacted the decision of participants to visit a dentist (18). For instance, 35% of individuals without dental insurance reported not visiting a dentist due to high treatment costs (8).

#### 3.4.2 Structural and logistical barriers

Distance was also observed to be a significant barrier in 28% of the included studies along with transportation availability [*n* = 1; (20)], long waiting periods to schedule appointments [*n* = 1; (22)], lack of appointments [*n* = 1; (22)], and availability of dental clinics [*n* = 1; (20)].

According to Croucher and Sohanpal (22), distance as a factor influenced dental visits, with participants only attending the nearest dental facilities. The lack of adequate transportation further restricted participants' access to oral care services (20). For example, 20% of respondents reported a lack of transportation to access dental care facilities (20). Furthermore, the availability of appointments also significantly influenced the access to dental care facilities. Croucher and Sohanpal (22) reported that participants expressed concerns about extended wait times while scheduling routine appointments. Although emergency appointments were accessible, participants reported waiting for approximately two months for routine checkups, exacerbating their unmet oral health needs. Additionally, the limited number of dental clinics further contributed to reduced access to dental care. Stormon et al. (20) reported that 25% of participants avoided dental visits due to a lack of facilities in their neighbourhood.

#### 3.4.3 Psychological barriers

Dental anxiety or fear was also a significant factor influencing respondents' decisions to accept treatment. Croucher and Sohanpal (22) observed that participants reported anxiety associated with treatment costs which was heightened by the lack of transparency and consistency in fee structures across different dental practices. Moreover, anxiety related to the acceptance of treatment from a dentist of the opposite gender was reported as a concern among participants, further contributing to apprehension (22). Consequently, these factors were associated with a reduced likelihood of accessing dental care. Potential strategies to mitigate dental anxiety and improve accessibility may include enhancing transparency in treatment plans and fee structures, as well as fostering a more welcoming and supportive clinical environment through improved patient-dentist interactions.

### 3.5 Risk of bias assessment

A summary of the risk of bias assessment is presented in Table 2, which employs colour coding where green denotes a low risk of bias and red indicates a high risk of bias. The assessment, utilizing the CLARITY Group's Risk of Bias Instrument for Cross-Sectional Surveys of Attitudes and Practices (2021), elucidated significant variability in the reliability of reported outcomes. Among the seven studies evaluated, four (8, 18, 19, 22) demonstrated a high risk of bias in one or more of the domains of the instrument. This elevated risk was primarily attributed to substantial missing data, low response rates, and reliance on volunteer sampling, all of which may limit the generalizability of the findings. Conversely, three studies (20, 21, 23) were determined to have an overall moderate to low risk of bias, as they employed rigorous methodological approaches, including random sampling strategies and the use of validated survey instruments, which resulted in low missing data and an adequate response rate.

**Table 2:** Ratings of included cross-sectional studies using CLARITY group's risk of bias instrument for cross-sectional surveys of attitudes and practices.

Author(s), year	Is the source population representative of the population of interest?	Is the response rate adequate?	Are there little missing data?	Is the survey clinically sensible?	Is there any evidence for the reliability and validity of the survey instrument?
Aday and Forthofer, 1992 (18)	Probably yes	Definitely yes	Definitely no	Probably yes	Probably no
Chattopadhyay et al., 2003 (8)	Probably yes	Probably no	Definitely yes	Definitely yes	Probably yes
Cohen et al., 2011 (19)	Probably yes	Definitely yes	Definitely yes	Probably no	Definitely yes
Croucher and Sohanpal, 2006 (22)	Definitely no	Definitely yes	Definitely yes	Probably yes	Probably yes
Manji et al., 1989 (23)	Probably yes	Definitely yes	Probably yes	Probably yes	Probably yes
Smith and Szuster, 2000 (21)	Probably yes	Definitely yes	Probably yes	Definitely yes	Definitely yes
Stormon et al., 2019 (20)	Probably yes	Definitely yes	Definitely yes	Definitely yes	Probably yes

## 4 Discussion

This review sought to assess the breadth and scope of literature addressing the barriers to oral health care access and the utilization of oral health services among socially marginalized youth on a global scale. Despite the increased developments in research and efforts directed toward promoting the health of equity-seeking populations, substantial effort is still required to attain health equity for socially marginalized youth. This vulnerable population has limited access to oral healthcare and insurance coverage which exacerbates adverse health outcomes, including mental illnesses such as depression and anxiety, as well as chronic diseases like diabetes (24). To our knowledge, this is the first scoping review that examines the literature on oral health care across multiple socially marginalized youth groups, and it found that oral health research particular to this vulnerable population is limited.

Our results highlight poor oral health outcomes among socially marginalized youth due to unmet oral health needs. In four of seven studies, participants reported conditions such as decayed and missing teeth, infections, and periodontal issues (19–21, 23). These adverse outcomes may stem from limited awareness of preventive oral health measures and available services (25, 26). Consequently, there is a need for the development of targeted educational interventions aimed at improving the oral health of socially marginalized youth. Public health interventions tailored to this group could enhance awareness about the importance of oral health. Our results highlighted that unmet dental treatment needs were strongly associated with access to dental care facilities, with socioeconomic factors such as cost and insurance coverage, significantly influencing service utilization. Therefore, it is imperative for policymakers to prioritize the mitigation of these social determinants to improve access to dental services. Interventions such as income-based subsidies could play a critical role in improving both the affordability and accessibility of dental care services (27).

Among socially marginalized youth, our studies identified groups such as refugees and other ethnic minorities with severe dental problems and unmet needs when compared to their counterparts. This highlights the intersectionality of various social determinants of health with unmet oral health needs and dental service utilization. According to Crenshaw (28), intersectionality is described as the interaction of an individual's social attributes such as race, ethnicity, age, gender, education, socioeconomic status, and sexual orientation or gender identity which collectively determine their social identity. However, limited knowledge exists regarding the interaction of these social determinants and their cumulative impact on oral health and access to care particularly in this population (29). Existing evidence indicates that the cumulative effect of social determinants substantially increases the risk of unmet dental needs and limited access to dental care. For instance, Anticona et al. (29) reported a higher prevalence of unmet dental needs among immigrants with low education and income compared to non-immigrants with higher education and income. Similarly, Bastos et al. (30) identified significantly higher odds of avoiding dental visits among Black men living below the poverty line when compared to White men living above the poverty line. Consequently, it is imperative to investigate the intersectional experiences of individuals in dental care, considering factors such as ethnicity, socioeconomic status, and religious beliefs (31). Adopting an intersectionality framework could enhance the understanding of health inequities. This approach could facilitate identifying populations that are most susceptible to barriers in utilizing dental services causing these populations to disengage from care.

Our review also highlighted the low utilization rates of dental care services in this population (8, 18, 20). This low utilization of services can be attributed to a lack of insurance, as unemployed individuals do not have access to employer-sponsored private insurance (32). Furthermore, inadequate education among low-income individuals may lead to a lack of awareness and knowledge about preventive health services, thereby limiting access to dental care (33). Other factors such as age, gender, education, and occupation can be associated with low dental service utilization. For example, Rahman (34) reported that individuals with lower educational levels had a reduced likelihood of utilizing dental care compared to those with higher educational levels. Similarly, Kim et al. (35) reported that individuals with only an elementary-level education or lower were less likely to utilize dental services, resulting in unmet needs, compared to individuals with university-level education or higher. These findings underscore the barriers socially marginalized youth experience while accessing oral health services. In addition to these social barriers, Griner et al. (36) also identified various psychological barriers restricting accessibility to oral care among socially marginalized youth.

Our findings underscore that anxiety and fear experienced by participants substantially influenced their willingness to seek dental care (22). A significant factor contributing to this anxiety was identified as discomfort with receiving treatment from practitioners of the opposite gender than that of the patient (22). Additionally, Griner et al. (36) indicate that discrimination based on gender, race, or ethnicity may further heighten anxiety and fear among youth, thereby restricting their access to dental care services.

Our findings corroborate that cost is a significant factor for youth in avoiding dental care services (8, 19, 20, 22). This issue is heightened by factors such as the lack of insurance and homelessness (8, 20). For instance, Stormon et al. (20) reported that approximately 64% of homeless youth avoided visiting dental care facilities due to high costs. Consequently, our review indicated that participants often accessed dental care services for emergency purposes rather than preventive measures (19). Additionally, distance and lack of transportation present significant barriers to accessing care (20, 22, 23). The lack of adequate transportation increases inaccessibility issues among socially marginalized youth who live far from dental care facilities. Therefore, to mitigate barriers to oral health services, policymakers should consider implementing subsidies that facilitate dental care access for equity seeking communities. This approach is particularly crucial in regions where oral health services are predominantly privately administered, such as in Canada and the United States (37).

Regarding study designs, most of the studies exhibited limited sample sizes, raising concerns regarding the generalizability of their findings. However, recruitment challenges within equity seeking populations may have contributed to these sample sizes (38–40). Gatlin and Johnson (39) highlight the difficulties in data collection among equity seeking communities such as immigrants, Indigenous individuals, transgender individuals, and racial and ethnic minorities. Researchers often encounter issues such as mistrust toward health-related research, challenges in conveying the benefits of participation, time constraints, fear of public exposure, cultural beliefs that discourage participation, and low literacy levels (39). Additionally, few of the identified studies used a non-binary form of gender expression and lacked inclusion of gender and sexual minorities (LGBT+) (20–22). Significant gaps exist in understanding the oral health of LGBT+ youth, with very limited to no evidence on unmet oral health needs and patterns of dental service utilization within this population (14). Therefore, to address these challenges and enhance recruitment, strategies such as engaging community navigators or providing financial compensation and gift vouchers could be implemented to achieve a representative sample population (40–42).

This review has several limitations. A notable limitation of this review is the variability in the age ranges reported across the included studies. The literature suggests that youth cannot be accurately defined by specific age brackets. Therefore, achieving consistency in age ranges among the included studies proved challenging. Although most of the included studies stratified participants by age, two of the seven studies did not implement age-based stratification (18, 22). Consequently, the findings reported are not age-specific but rather generalizable to the wider age spectrum (>18 years, and 18–40 years, respectively). Nonetheless, despite this broad age spectrum, the findings of the two studies provide crucial insight into the target population. Furthermore, the limited number of studies included in this review highlights a critical research gap, underscoring the need for a more tailored approach that specifically addresses the oral health needs of socially marginalized youth. Developing such an approach is crucial for understanding the impact of marginalization on this demographic and its effects on access to oral healthcare.

Another significant limitation of this review is the focus on marginalization as a collective phenomenon, without adequately addressing the distinct environmental challenges experienced by specific subgroups of socially marginalized youth. Subpopulations such as refugees, racial and ethnic minorities, LGBTQ+ individuals, and homeless youth likely encounter unique environmental barriers that influence both their access to and utilization of dental care. To address this gap, future research should conduct subgroup-specific analyses and propose targeted, evidence-based interventions tailored to the particular challenges faced by each group. Such an approach could yield more precise insights and strategies for effectively addressing barriers unique to these populations. Furthermore, there exists an absence of information regarding the influence of national or regional policies on the accessibility of dental services for socially marginalized youth. Subsequent studies should examine the impact of existing policies, evaluating their effectiveness in enhancing access to dental care for marginalized populations and identifying potential gaps that require further attention and intervention.

Additional limitations of this review are the restricted geographic scope of the included studies, which may limit the generalizability of the findings to other global contexts. Also, the search strategy was limited to English-language papers, thereby excluding research published in other languages. However, the extent of relevant studies in languages other than English remains unclear.

## 5 Conclusion

Our review identified a significant research gap concerning the unmet oral health needs and barriers to accessing dental services among socially marginalized youth. However, limited available evidence highlights poor oral health outcomes within this population, with a high prevalence of unmet needs, including dental caries and periodontal diseases. Furthermore, the barriers experienced by these individuals significantly restrict their utilization of dental care services. Although some studies utilized validated measures (e.g., the DMFT index) to assess oral health, our findings highlight significant limitations, including small sample sizes and lack of varied study designs. Despite these limitations, this review provides a comprehensive overview of the available evidence concerning the barriers to oral health services for socially marginalized youth, identifies gaps in the literature, and suggests directions for future research. Notably, more robust and representative research is required to gain a deeper understanding of the oral health status of marginalized youth. Future efforts by oral health advocates should focus on ensuring that socially marginalized youth populations can both access and benefit from oral health care services. Potential intervention strategies could include increasing awareness of the importance of oral health through the distribution of informational materials, such as brochures and leaflets, and organizing oral health awareness programs in educational institutions and community settings. Additionally, engaging community healthcare providers and dental professionals in developing tailored dental education resources and programs may enhance the effectiveness of these initiatives.

## Appendix A

**Table:**

	Database			
Concept	MEDLINE (Ovid)	Embase	Scopus	Cochrane library
Oral Health/Dental Health	“oral health”.tw,kf. or “oral care”.tw,kf. or dental.tw,kf. or oral health or exp dental care or [exp delivery of health care and (dentistry or dental).tw,kf.]	“oral health”.tw,kf. or “oral care”.tw,kf. or dental.tw,kf. or dental health or [health care delivery and (dentistry or dental).tw,kf.]	((title-abs-key (“oral health”)) or (title-abs-key (“oral care”)) or (title-abs-key (dental))) or ((title-abs-key (“dental care”)) or ((title-abs-key (“delivery of health care”)) and (title-abs-key ((dentistry or dental)))))	(“oral health”):ti,ab,kw or (“oral care”):ti,ab,kw or (“dental care”):ti,ab,kw or [oral health] or [dental care](exploded) or {[delivery of health care] (exploded) and (dentistry or dental):ti,ab,kf}
Youth	“young adult”.tw,kf. or youth.tw,kf. or “young individual*”.tw,kf. or “young people”.tw,kf. or “young person*”.tw,kf. or teen*.tw,kf. or adolescen*.tw,kf. or young adult or adolescent/	“young adult”.tw,kf. or youth.tw,kf. or “young individual*”.tw,kf. or “young people”.tw,kf. or “young person*”.tw,kf. or teen*.tw,kf. or adolescen*.tw,kf. or young adult or adolescent/	(title-abs-key (“young adult”)) or (title-abs-key (youth)) or (title-abs-key (“young individual*”)) or (title-abs-key (“young people”)) or (title-abs-key (“young person”)) or (title-abs-key (teen*)) or (title-abs-key (adolescen*))	(“young adult”):ti,ab,kw or (youth):ti,ab,kw or (“young individual”):ti,ab,kw or (“young person”):ti,ab,kw or (teen*):ti,ab,kw or (adolescen*):ti,ab,kw or [young adult] or [adolescent]
Marginalization	Marginali*.tw,kf. or “socially disadvantage*”.tw,kf. or disadvantaged.tw,kf. or minorit*.tw,kf. or minority groups or ethnic minorities or “sexual and gender minorities” or social marginalization/	Marginali*.tw,kf. or “socially disadvantage*”.tw,kf. or disadvantaged.tw,kf. or minorit*.tw,kf. or social exclusion or minority group or “sexual and gender minority”/	(title-abs-key (marginali*)) or (title-abs-key ((“socially disadvantag*”))) or (title-abs-key (disadvantaged)) or (title-abs-key ((minorit*))) or (title-abs-key ((“social marginalization”))) or (title-abs-key ((“minority groups”)))	(marginali*):ti,ab,kw or (socially disadvantaged): ti,ab,kw or (disadvantaged):ti,ab,kw or (minorit*):ti,ab,kw or [social marginalization] or [minority groups] or [ethnic and racial minorities] or [sexual and gender minorities]
Barriers to care	Barrier*.tw,kf. or (access adj3 care).tw,kf. or (access adj health).tw,kf. or Health services accessibility/	Barrier*.tw,kf. or (access adj3 care).tw,kf. or (access adj health).tw,kf. or Health care access/	(title-abs-key (barrier*)) or (title-abs-key (access*))	(barrier*):ti,ab,kw or (access to care):ti,ab,kw or [health services accessibility]
Linking concepts	1 AND 2 AND 3 AND 4 *N* = 152	1 AND 2 AND 3 AND 4 *N* = 105	1 AND 2 AND 3 AND 4 *N* = 219	1 AND 2 AND 3 AND 4 *N* = 8
