# Supplementary figures and images for: Unmet oral health needs and barriers to dental services among socially marginalized youth: a scoping review
Source: Front Oral Health. 2025 Mar 12;6:1521753. doi: 10.3389/froh.2025.1521753 (PMC11937129; doi:10.3389/froh.2025.1521753)

# **Supplement 2**

## Data extraction tool


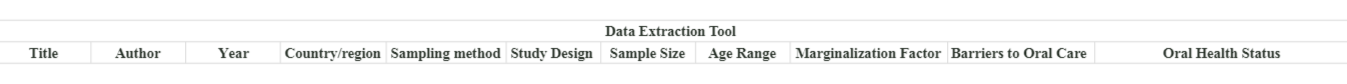


## Final data extraction tool


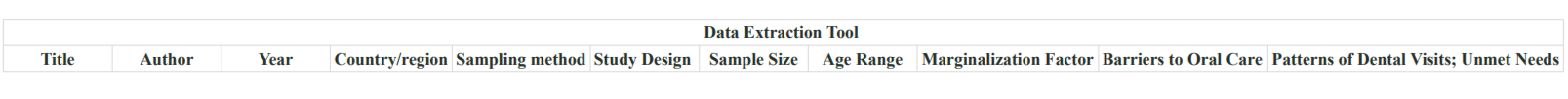

Supplement: Supplementary file 2 [file Supplementaryfile2.docx]
